# Molecular Hallmarks of Ischemia with Non-Obstructive Coronary Arteries: The “INOCA versus Obstructive CCS” Challenge

**DOI:** 10.3390/jcm11061711

**Published:** 2022-03-19

**Authors:** Alice Bonanni, Alessia d’Aiello, Daniela Pedicino, Marianna Di Sario, Ramona Vinci, Myriana Ponzo, Pellegrino Ciampi, Denise Lo Curto, Cristina Conte, Francesco Cribari, Francesco Canonico, Giulio Russo, Rocco Antonio Montone, Carlo Trani, Anna Severino, Filippo Crea, Giovanna Liuzzo

**Affiliations:** 1 Department of Cardiovascular and Pulmonary Sciences, Catholic University of the Sacred Heart, 00168 Rome, Italy; bonanni.alice92@gmail.com (A.B.); alessia.daiello@gmail.com (A.d.); mariannadisario@gmail.com (M.D.S.); ramona.vinci@unicatt.it (R.V.); myriana.ponzo@gmail.com (M.P.); pellegrino.ciampi.rc@gmail.com (P.C.); denise.locurto@virgilio.it (D.L.C.); cristinaconte93@gmail.com (C.C.); fracrib93@gmail.com (F.C.); giuliorusso.md@gmail.com (G.R.); roccoantonio.montone@policlinicogemelli.it (R.A.M.); carlo.trani@policlinicogemelli.it (C.T.); anna.severino1@unicatt.it (A.S.); filippo.crea@unicatt.it (F.C.); giovanna.liuzzo@gmail.com (G.L.); 2 Department of Cardiovascular Sciences, Fondazione Policlinico Universitario Agostino Gemelli IRCCS, 00168 Rome, Italy; fra.canonico1984@gmail.com

**Keywords:** non-obstructive CAD, ischemia with non-obstructive coronary artery (INOCA), chronic coronary syndromes (CCS), coronary microvascular dysfunction (CMD), biomarker, gene expression, precision medicine

## Abstract

Up to 4 million patients with signs of myocardial ischemia have no obstructive coronary artery disease (CAD). The absence of precise guidelines for diagnosis and treatment in non-obstructive CAD encourages the scientific community to fill the gap knowledge, to provide non-invasive and less expensive diagnostic tools. The aim of our study was to explore the biological profile of Ischemia with Non-Obstructive Coronary Arteries (INOCA) patients with microvascular dysfunction compared to patients presenting with obstructive chronic coronary syndrome (ObCCS) in order to find specific hallmarks of each clinical condition. We performed a gene expression array from peripheral blood mononuclear cells (PBMCs) isolated from INOCA (*n* = 18) and ObCCS (*n* = 20) patients. Our results showed a significantly reduced gene expression of molecules involved in cell adhesion, signaling, vascular motion, and inflammation in INOCA as compared to the ObCCS group. In detail, we found lower expression of Platelet and Endothelial Cell Adhesion Molecule 1 (CD31, *p* < 0.0001), Intercellular Adhesion Molecule-1 (ICAM1, *p* = 0.0004), Tumor Necrosis Factor (TNF *p* = 0.0003), Transferrin Receptor (TFRC, *p* = 0.002), and Vascular Endothelial Growth Factor A (VEGFA, *p* = 0.0006) in the INOCA group compared with ObCCS. Meanwhile, we observed an increased expression of Hyaluronidase (HYAL2, *p* < 0.0001) in INOCA patients in comparison to ObCCS. The distinct expression of molecular biomarkers might allow an early and non-invasive differential diagnosis between ObCCS and INOCA, improving clinical management and treatment options, in the era of personalized medicine.

## 1. Introduction

Ischemia with Non-Obstructive Coronary Arteries (INOCA) is far from an uncommon condition. Indeed, up to 50% of patients undergoing diagnostic coronary angiography for typical chest pain have no obstructive coronary artery disease (CAD) [1]. Manifold mechanisms seem to contribute to the INOCA condition, including coronary microvascular dysfunction (CMD) and coronary vasospasm, both alone or combined [2,3,4]. Traditional atherosclerosis risk factors such as aging, hypertension, diabetes mellitus, and dyslipidaemia are strongly associated with increased risk for CMD [5]. Nevertheless, traditional risk factors are not always present in CMD, and novel risk markers such as those associated with endothelial dysfunction and inflammation may contribute [6,7].

Despite the reassuring coronary aspect, increasing evidence demonstrates that the prognosis associated with INOCA is not benign. A large portion of patients presenting chest discomfort, shortness of breath, and normal angiography are discharged with a diagnosis of non-cardiac chest pain. For these patients, a cardiac event (including acute coronary syndrome and repeat cardiovascular procedures) or a hospitalization for heart failure with preserved ejection fraction (HFpEF) may occur within 10 years [8,9,10,11]. Alongside this, the presence of no obstructive CAD at coronary angiography may be associated with diagnostic and therapeutic uncertainty, resulting often in undertreatment despite the fact that the knowledge about this condition is rapidly increasing [12].

Although multiple noninvasive techniques (including transthoracic Doppler echocardiography, myocardial contrast echocardiography, positron emission tomography, magnetic resonance imaging, and single-photon emission computed tomography) are now available to help in detecting ischemia in INOCA, diagnosis still relies on coronary angiography, exposing the patients to potentially avoidable additional risks related to the procedure. In this scenario, defining a biological signature might be useful to reduce invasive and expensive tests, to predict risk, to improve our INOCA pathophysiology knowledge and to propose novel tailored therapies.

## 2. Materials and Methods

### 2.1. Study Population

We prospectively enrolled 38 consecutive patients admitted to our cardiovascular care unit to undergo coronary angiography because of stable, chronic symptoms suggesting ischemic heart disease. According to the result of coronary angiography, patients have been divided into two groups: 18 patients presenting with INOCA and 20 with obstructive chronic coronary syndrome (ObCCS).

### 2.2. Enrolment Criteria

INOCA patients were defined as follows:

– Stable, chronic symptoms suggesting ischemic heart disease such as chest discomfort with both typical angina pectoris or atypical features in terms of location, quality, and in-citing factors.
– Objective evidence of myocardial ischemia from the electrocardiogram (ECG) or a cardiac imaging study (echocardiography, nuclear imaging, magnetic resonance imaging, or spectroscopy) at rest or during stress (exercise or pharmacological), without the rise of myocardial injury biomarkers.
– Absence of flow-limiting obstruction by coronary angiography as defined by any epicardial coronary artery diameter reduction ≥50% or fractional flow reserve <0.8.
– Evidence of angina with a micro-vascular origin, identified during intracoronary infusion of acetylcholine with typical ischemic ST-segment changes without epicardial coronary constriction (<90% re-duction) in coronary artery diameter [13]. As described elsewhere, functional mechanisms responsible for CMD may be related to the presence of an impaired dilation (vasodilator abnormalities, most often detected as reduced coronary flow reserve -CFR-), an increased constriction of coronary micro vessels (microvascular spasm) or a combination of both mechanisms. Our population definitely belongs to the latter group [4].

On the other hand, ObCCS patients were defined as follows:

– Symptoms of stable effort angina lasting more than 12 months.
– Obstructive CAD confirmed at the coronary angiography [10].

Exclusion criteria were:

– Age >85 years.
– Evidence of infectious diseases, malignancies, immunologic or haematological disorders.
– Allergic disorders.
– Severe chronic HF (left ventricular ejection fraction -LVEF < 35%).
– Treatment with anti-inflammatory drugs other than low-dose aspirin.
– Chronic kidney disease stage 4 (glomerular filtration rate -GFR < 30 mL/min).

### 2.3. Ethical Clearance

All individuals gave their written informed consent. The Ethics Committee of the Fondazione Policlinico Universitario “A. Gemelli” IRCCS—Catholic University of Sacred Heart of Rome approved the study.

### 2.4. Blood Sampling and PBMC Isolation

In addition, 30 Cc of venous blood samples were collected in vacuettes with EthylenDiaminoTetracetyc Acid (EDTA) through venipuncture at the time of patient enrollment. We isolated peripheral blood mononuclear cells (PBMCs) from whole blood by the density gradient centrifugation method (Lympholyte^®^-H Cell Separation Media, CEDARLANE, Burlington, ON, Canada). We layered blood over Ficoll with a ratio of 1:2 and centrifuged it at 1100× *g* for 25 minutesat room temperature, without break. PBMCs were then washed, resuspended in Dulbecco’s phosphate-buffered saline (DPBS Invitrogen, Carlsbad, CA, USA), and aliquoted (5 × 10^6^ cells/vial). Aliquots of PBMCs were centrifuged at 1600× *g* for 10 min. Finally, pellets were dried and stored at −80 °C.

### 2.5. RNA Extraction and Retro-Transcription

Total RNA was extracted from stored PBMCs with RNeasy Plus Extraction Kit (QIAGEN GmbH, Hilden, Germany) and subjected to qualitative and quantitative control using a Multiskan™ GO Microplate Spectrophotometer (Thermo Fisher Scientific, Waltham, MA, USA). RNA reverse transcription in first-strand cDNA was done with iScript cDNA synthesis kit (Bio-Rad, Hercules, CA, USA). Obtained cDNAs were stored at −20 °C for scheduled molecular investigations. 

### 2.6. Prime PCR Arrays

We selected markers of inflammation, oxidative stress, cell adhesion, vasoconstriction, apoptosis and extracellular matrix (ECM) remodeling. We designed a custom 96-well plate PrimePCR (Bio-Rad, Hercules, CA, USA), including the following gene: ADAMTS13; ALOX5; B2M; CD44; EDN1; GPX1; HBA1; HYAL2; ICAM1; LGALS8; MMP1; MMP2; MMP9; NOS3; PI16; PLA2G7; SOD1; TFRC; TIMP1; TNF; VCAM1; VEGFA. Table 1 indicates gene nomenclature and function.

### 2.7. Networking Analysis

We used STRING Database, version 11.5, (Search Tool for the Retrieval of Interacting Genes/Proteins; http://string-db.org [viewed on 10 June 2021]) as a pre-computed database for the analysis of protein–protein networks. The associations originate from high-throughput experimental data, mining of literature, databases and analyses of co-expressed genes. STRING applies a particular scoring to generate a single confidence score per prediction [14].

### 2.8. Gene Expression on Pooled cDNA

We performed the gene expression array on two groups of pooled cDNAs from PBMCs of INOCA (*n* = 10) and ObCCS patients (*n* = 10).

As described by the manufacturer, all PrimePCR arrays were designed following strict guidelines on maximum transcript coverage, minimal overlap with known SNPs, and spanning large introns where possible. In addition, they have all been validated passing stringent quality controls. In accordance with the MIQE guidelines [15], there was full transparency on the performance of every PrimePCR assay in the form of a standardized specification and validation sheet that can be found on the Bio-Rad website, www.bio-rad.com/PrimePCR [viewd on 29 June 2021].

### 2.9. Validation of Gene Expression

We designed primers from nucleotide sequences identified using NCBI BLAST (http://blast.ncbi.nlm.nih.gov/Blast.cgi [viewed on 6 September 2021]), and they were ordered from BioFab Research Srl (Rome, Italy) with their certificates of analysis. All of the genes listed above have been validated in the two groups of INOCA (*n* = 18) and ObCCS (*n* = 20) patients. MMP2 and VCAM1 genes have been excluded due to the elevated threshold cycle (>36–38 *C_T_*). We chose beta 2-microglobulin (B2M) as a housekeeping reference gene. All RT-qPCRs were run in duplicate and performed using CFX96™ Touch Real-Time PCR Detection System (Bio-Rad, Hercules, CA, USA). Analyses were performed through Bio-Rad CFX Maestro 1.1 Software. Relative gene expressions were then calculated using the 2^−∆∆CT^ method. 

DNA oligonucleotide sequences are listed in Table 2.

### 2.10. Statistical Analysis

The distribution of continuous variables was assessed through the Shapiro–Wilk test and described as mean and standard deviation (mean ± SD) for normally distributed data and as median and interquartile range (IQR) for not normally distributed data. To analyse the means of the two groups, since values did not have a normal distribution, a Mann–Whitney test was used. Meanwhile, to compare the means of two groups with continuous values following a normal distribution, an unpaired *t*-test with Welch’s correction was used. A two-tailed *p*-value < 0.05 was considered statistically significant. Statistical analysis was performed with GraphPad Prism version 8.0.2 for Windows, (GraphPad Software, La Jolla, CA, USA) and with STATA IC 16.1 (StataCorp LLC, TX, USA). Receiver operating characteristic (ROC) analysis has been used to evaluate diagnostic accuracy and to select the optimal threshold value, balancing the intrinsic compromises that stand between sensitivity and sensitivity [16].

## 3. Results

We evaluated demographic data, classical cardiovascular risk factors, history of previous acute coronary syndromes, previous coronary revascularization procedures, ventricular function, and medical treatments. Characteristics of the study population are reported in Table 3.

**Figure 1:**
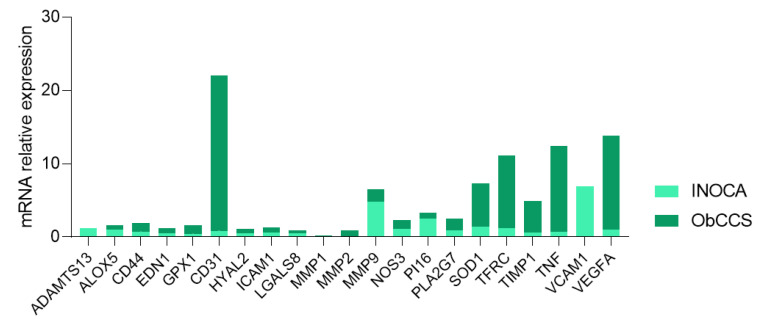
Histogram showing expression of pooled PBMC-cDNAs of INOCA (*n* = 10) and ObCCS (*n* = 10) patients. INOCA = ischemia with non-obstructive coronary artery; ObCCS = obstructive chronic coronary syndrome; PBMC = peripheral blood mononuclear cell; ADAMTS13 = ADAM Metallopeptidase with Thrombospondin Type 1 Motif 13; ALOX5 = Arachidonate 5-Lipoxygenase; CD31 = Platelet And Endothelial Cell Adhesion Molecule 1; CD44 = Hyaluronan receptor; EDN1 = Endothelin 1; GPX1 = Glutathione peroxidase 1; HYAL2 = Hyaluronidase 2; ICAM1 = Intercellular Adhesion Molecule 1; LGALS8 = Galectin 8; MMP1 = Matrix metalloproteinase 1; MMP2 = Matrix metalloproteinase 2; MMP9 = Matrix metalloproteinase 9; NOS3 = Endothelial nitric oxide synthase; PI16 = Peptidase Inhibitor 16; PLA2G7 = Phospholipase A2 Group VII; SOD1 = Superoxide dismutase 1; TFRC = Transferrin Receptor; TIMP1 = TIMP Metallopeptidase Inhibitor 1; TNF = Tumor Necrosis Factor; VCAM1 = Vascular cell adhesion molecule 1; VEGFA = Vascular endothelial growth factor A.

We conducted a custom PrimePCR array, investigating 21 genes (Table 2) in 10 INOCA and 10 ObCCS patients pooled cDNAs (Figure 1).

**Figure 2:**
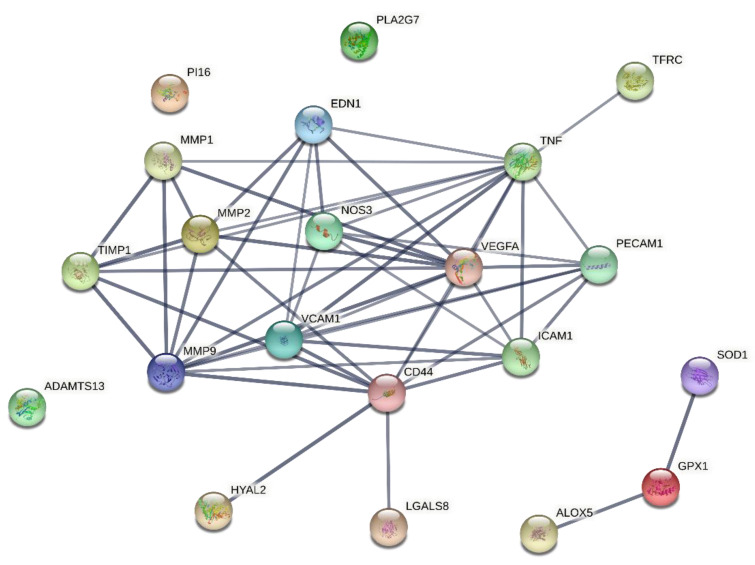
Protein–protein association network visualized by STRING. Each node represents all the proteins produced by a single, protein-coding gene locus, while each edge represents the number of protein–protein associations. Only interactions with a high confidence score of ≥0.7 are shown. The color saturation of the edges represents the confidence score of the functional association [17]. ADAMTS13 = ADAM Metallopeptidase with Thrombospondin Type 1 Motif 13; ALOX5 = Arachidonate 5-Lipoxygenase; CD31 = Platelet And Endothelial Cell Adhesion Molecule 1; CD44 = Hyaluronan receptor; EDN1 = Endothelin 1; GPX1 = Glutathione peroxidase 1; HYAL2 = Hyaluronidase 2; ICAM1 = Intercellular Adhesion Molecule 1; LGALS8 = Galectin 8; MMP1 = Matrix metalloproteinase 1; MMP2 = Matrix metalloproteinase 2; MMP9 = Matrix metalloproteinase 9; NOS3 = Endothelial nitric oxide synthase; PI16 = Peptidase Inhibitor 16; PLA2G7 = Phospholipase A2 Group VII; SOD1 = Superoxide dismutase 1; TFRC = Transferrin Receptor; TIMP1 = TIMP Metallopeptidase Inhibitor 1; TNF = Tumor Necrosis Factor; VCAM1 = Vascular cell adhesion molecule 1; VEGFA = Vascular endothelial growth factor A.

A STRING graphic has been developed to underline the protein–protein association between the pathways taken into consideration in the study. In total, 21 genes were analysed using STRING. The network analysis revealed 21 nodes, 51 number of edges, an average node degree of 4.86, while the protein–protein interaction (PPI) enrichment *p*-value was <1.0 × 10^−16^, and the average local clustering coefficient was 0.684 (Figure 2).

Gene expression validation has been conducted on 18 INOCA and 20 ObCCS patients. MMP2 e VCAM1 genes were excluded due to a cycle threshold (Ct) > 36.5 cycles, being, therefore, non-reliable; among the remaining 19 genes, six were statistically significant different between INOCA and ObCCS patients. 

**Figure 3:**
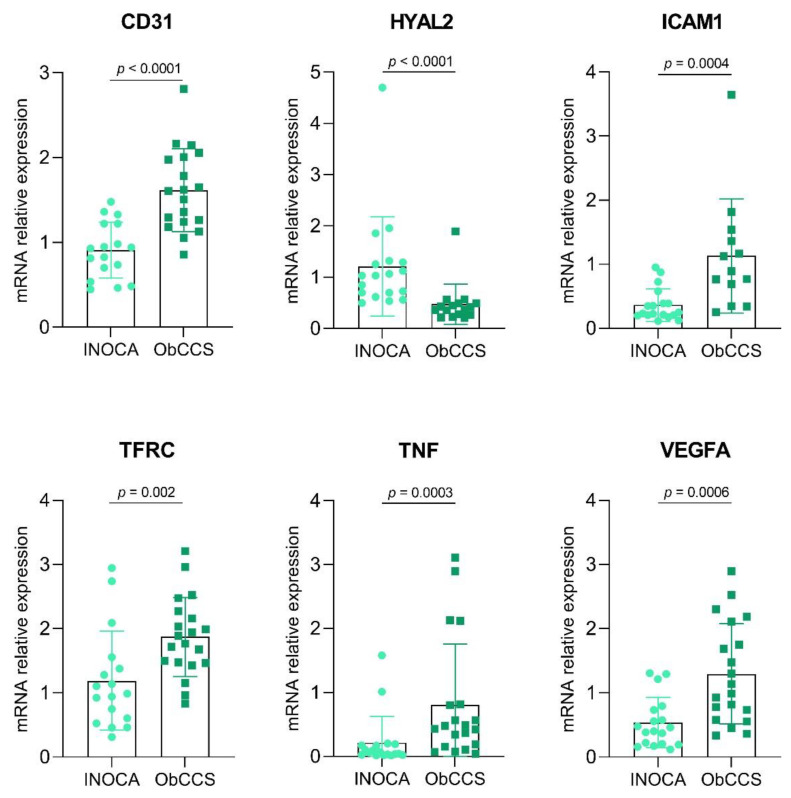
Histograms showing expression of PBMC-cDNAs of INOCA (*n* = 18) and ObCCS (*n* = 20) patients. INOCA = ischemia with non-obstructive coronary artery; ObCCS = obstructive chronic coronary syndrome; PBMC = peripheral blood mononuclear cell; CD31 = Platelet And Endothelial Cell Adhesion Molecule 1; HYAL2 = Hyaluronidase 2; ICAM1 = Intercellular Adhesion Molecule 1; TFRC = Transferrin Receptor; TNF = Tumor Necrosis Factor; VEGFA = Vascular endothelial growth factor A.

CD31, ICAM1, TFRC, TNF and VEGFA gene expressions were significantly lower in INOCA as compared to ObCCS patients (mean ± SD: for CD31 0.91 ± 0.33 vs. 1.62 ± 0.49, *p* < 0.0001. Median, IQR: for ICAM1 0.24, 0.24 vs. 0.89, 0.93; *p* = 0.0004; for TFRC 0.99, 0.90 vs. 1.83, 0.78; *p* = 0.002; for TNF 0.06, 0.15 vs. 0.46, 0.65; *p* = 0.0003; for VEGFA 0.43, 0.55 vs. 1.06, 1.4; *p* = 0.0006) as shown in Figure 3. Meanwhile, HYAL2 was more expressed in INOCA patients compared to ObCCS patients (Median, IQR: HYAL2 1.03, 0.62 vs. 0.39, 0.22; *p* < 0.0001) (Figure 3).

**Figure 4:**
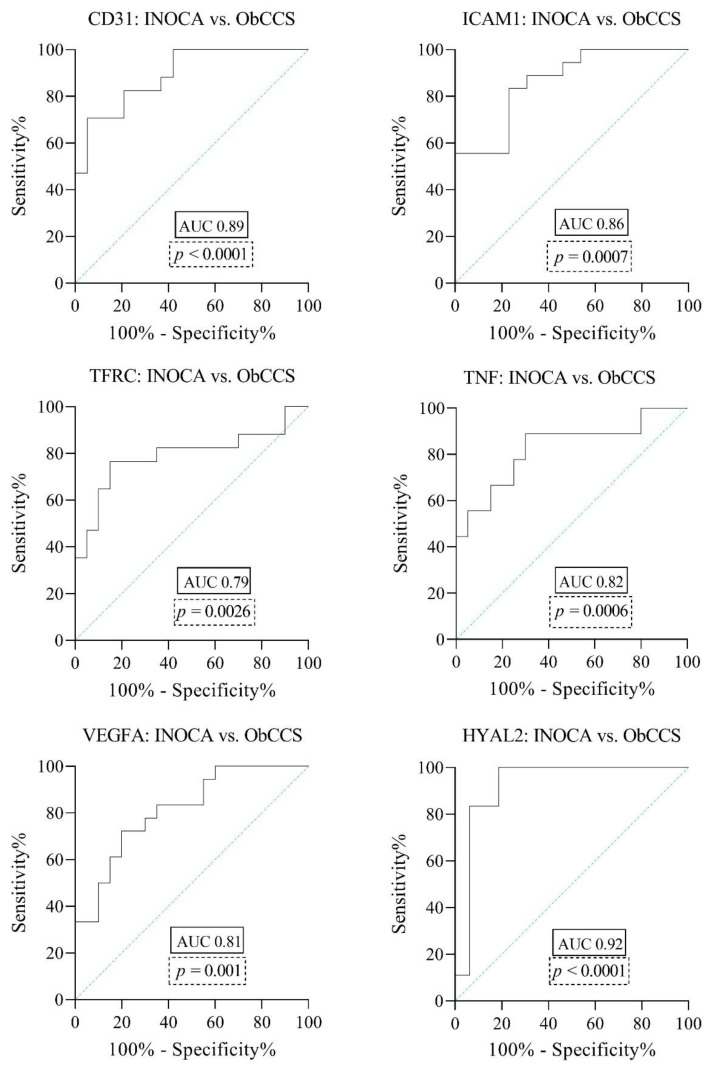
Receiver operating characteristic (ROC) curves for the prediction of INOCA/ObCCS based on the gene expression of CD31, ICAM1, TFRC, TNF, VEGFA, HYAL2. The area under the curve (AUC) equals 0.5 when the ROC curve corresponds to random chance and 1.0 for perfect accuracy. An AUC greater than 0.9 has high accuracy, while 0.7–0.9 indicates moderate accuracy and 0.5–0.7 low accuracy. CD31 = Platelet And Endothelial Cell Adhesion Molecule 1; HYAL2 = Hyaluronidase 2; ICAM1 = Intercellular Adhesion Molecule 1; TFRC = Transferrin Receptor; TNF = Tumor Necrosis Factor; VEGFA = Vascular endothelial growth factor A.

Finally, for the molecules with a significantly different gene expression between INOCA and ObCCS, receiver operating characteristic (ROC) analyses showed an Area Under the Curve (AUC) indicating high or moderate accuracy as biomarkers, with the following values: 0.89 for CD31 (*p* < 0.0001), 0.86 for ICAM1 (*p* = 0.0007), 0.79 for TFRC (*p* = 0.0026), 0.82 for TNF (*p* = 0.0006), 0.81 for VEGFA (*p* = 0.001) and 0.92 for HYAL2 (*p* < 0.0001) (Figure 4). 

Given the higher expression of HYAL2 in INOCA patients if compared with ObCCS and given the AUC values indicating the highest accuracy among the selected biomarkers, we defined the HYAL2 threshold value of 0.5896 with a sensitivity of 100% and a specificity of 93.75% to differentiate INOCA patients (HYAL2 gene expression values ≥ 0.5896) from ObCCS patients (HYAL2 gene expression values < 0.5896). Table 4 shows the detailed report of sensitivity and specificity for different HYAL2 gene expression cut-offs.

## 4. Discussion

To the best of our knowledge, this is the first study investigating gene expression in two classes of patients, ObCCS and INOCA, characterized by overlapping clinical presentation in spite of different angiographic findings. In particular, our data show significant differences in gene expression between these two populations: INOCA patients have decreased expression of genes involved in inflammatory pathways, cell adhesion, and immune-mediated response (TNF, TFRC, ICAM1, CD31, VEGFA), together with an increased expression of HYAL 2, a gene implicated in extracellular matrix turnover and hyaluronan metabolism. Analyzing the role of those genes, they are involved in several functions and mechanisms underlying atherosclerosis in its different stages. TNF increases the expression of adhesion molecules, which in turn induce cell proliferation and migration leading to plaque growth and thickening [18]. VEGFA assists TNF in this latter mechanism by stimulating cell proliferation and supplying inflammatory cells [19,20]. In addition, adhesion molecules like CD31 and ICAM1 are key components for the immune-mediated response, allowing recruitment, migration, and entry of leukocytes at the level of the vessel wall lesions [21,22], which lead to the progression of the stenotic plaques [23]. Finally, transferrin receptor (TFRC) is a mediator of iron cellular uptake. The importance of iron uptake has become evident through in vivo studies in mice, which develop as a consequence of Trf1 gene inactivation severe pathologies, such as cardiomyopathies [24]. However, iron overload and deficiency have both been associated with cardiac disorders. Indeed, mitochondria can suffer from iron-mediated toxicity, which leads to impaired mitochondrial function, enhanced ROS production, and progression of inflammatory status [25,26]. Notably, only one gene of our array, HYAL2, originally involved in hyaluronan catabolism and glycocalyx impairment [27,28,29], displayed an increased expression in PBMCs of INOCA patients. HYAL2 has been studied in the context of shear stress and plaque erosion [27,30,31]; however, no study before has investigated HYAL2 in INOCA patients. The endothelium bears a crucial aspect in vascular tone modulation. Hence, shear stress induces HYAL mediated glycocalyx derangement that is strongly related to CMD onset and progression [32]. According to current evidence, multiple mechanisms might contribute to INOCA, including coronary microvascular dysfunction (CMD) and coronary vasospasm [4,5,33]. In most patients, chest pain is induced by myocardial ischemia resulting from CMD [34,35] defined as epicardial, microvascular endothelial or nonendothelial dysfunction that limits myocardial perfusion, most often detected as reduced coronary flow reserve (CFR). CMD may occur both in the presence and in the absence of obstructive CAD and myocardial diseases [36]. In general, CMD and obstructive CAD share the same risk factors such as aging, hypertension, diabetes mellitus, and dyslipidemia although traditional risk factors are not always present in CMD, and novel risk markers such as those associated with inflammation may contribute [2,7].

The main pathogenic mechanisms of CMD are represented by endothelial dysfunction, smooth muscle cell dysfunction, and vascular remodeling [2]. Endothelin-1 (EDN1) has been investigated as responsible for CMD and endothelial dysfunction [37,38]. However, in CMD, due to a low-grade inflammatory response, several inflammatory biomarkers have been involved (TNF, IL-6 and hs-CRP) [38,39,40,41]. This immune reaction, through the increase of chemokines and cell adhesion factors, such as TNF, VCAM, and ICAM1, drives and supports monocytes’ activation, migration, and extravasation. Additionally, wall shear stress (WSS) enhances this response through biochemical signals, initiated by endothelial cells, that modulates leukocytes’ adhesion, platelets’ activity, vascular tone, and endothelial impairment of oxidative balance (SOD1, GPX1, NOS3) [42,43]. Furthermore, Kong et al. showed that the alteration of the local flow could be a pro-inflammatory stimulus, leading to an increase of hyaluronidase (HYAL2) gene expression, possibly mediating endothelial dysfunction [27]. 

Another interesting hypothesis suggests that CMD might be a preliminary mechanism for epicardial lesion development [44], explaining, in this way, age discrepancies between INOCA and ObCCS patients justifying the late onset of epicardial lesions in ObCCS compared to INOCA group. In support of this hypothesis, recent data demonstrated that almost all patients with INOCA studied by intravascular ultrasound (IVUS) have some coronary atherosclerosis [45,46].

Nowadays, differentiating patients with evidence of inducible-ischemia and with ObCCS from those without represents a challenge for cardiologists and the only way to establish the final diagnosis is through invasive exams (coronary angiography including guidewire and vasoreactivity testing). In this perspective, the identification of gene expression cut-off obtained through blood samples could (1) contribute to better understand the underlying pathophysiological mechanisms of INOCA patients and (2) provide an important tool for the non-invasive diagnosis of INOCA and for the differential diagnosis with ObCCS. In this perspective, this study not only contributes to the knowledge of complex mechanism behind INOCA, but it also proposes a possible tool for the early differential diagnosis between INOCA and ObCCS. Further data are needed to better establish molecular pathways and underpinning mechanisms of INOCA and to further validate gene expression for the diagnostic work-up. 

### 4.1. Study Limitations

Our study includes a small number of patients. A much larger study should be conducted to assess a real signature and a possible cut-off to discriminate these CAD populations. For these reasons, HYAL2 cut-off values should be considered merely as indicatives and need to be further validated. Finally, no healthy controls were included in the study.

### 4.2. Clinical Translation

INOCA patients are associated with recurrent hospital admittance, increased incidence of cardiovascular events, and a poor quality of life [19]. Making an early differential diagnosis between patients with effort angina presenting with and without obstructive CAD through novel and effective molecular examinations might support this complex diagnostic pathway, paving the way toward a personalized clinical stratification. 

## 5. Conclusions

In our study, we describe for the first time an ex vivo molecular profile of INOCA condition, which could allow early identification of non-obstructive CAD. A biological signature in this clinical setting might represent an appealing, non-invasive diagnostic tool, supporting the use of angiography and imaging tests.

## Figures and Tables

**Table 1:** Gene nomenclature and function.

Gene Nomenclature	Gene Name	Principal Function
ADAMTS13	ADAM Metallopeptidase with Thrombospondin Type 1 Motif 13	von Willebrand factor cleavage
ALOX5	Arachidonate 5-Lipoxygenase	Leukotriene biosynthesis
CD31	Platelet And Endothelial Cell Adhesion Molecule 1	Ig-like adhesion molecule Leukocyte migration, angiogenesis, integrin activation, immunomodulation, mechanotrasduction
CD44	Hyaluronan receptor	Cell–cell interactions, cell adhesion and migration
EDN1	Endothelin 1	Vasoconstrictor
GPX1	Glutathione peroxidase 1	Redox-balancer
HYAL2	Hyaluronidase 2	Hyaluronan degradation
ICAM1	Intercellular Adhesion Molecule 1	Cell proliferation, differentiation, motility, trafficking, apoptosis and tissue architecture
LGALS8	Galectin 8	Cell–cell adhesion, cell–matrix interaction, growth regulation, apoptosis, and RNA splicing
MMP1	Matrix metalloproteinase 1	ECM and molecule degradation
MMP2	Matrix metalloproteinase 2	ECM and molecule degradation; remodeling of the vasculature, angiogenesis, tissue repair, inflammation, and atherosclerotic plaque rupture
MMP9	Matrix metalloproteinase 9	ECM and molecule degradation; leukocyte migration
NOS3	Endothelial nitric oxide synthase	Implication in vascular smooth muscle relaxation
PI16	Peptidase Inhibitor 16	Cardiomyocyte growth inhibition
PLA2G7	Phospholipase A2 Group VII	Platelet-activating factor (PAF) activity modulation
SOD1	Superoxide dismutase 1	Superoxide anion radical destruction
TFRC	Transferrin Receptor	Cell surface receptor for cellular iron uptake
TIMP1	TIMP Metallopeptidase Inhibitor 1	Cell proliferation and potential an anti-apoptotic function
TNF	Tumor Necrosis Factor	Cell apoptosis, proliferation, differentiation, lipid metabolism, and coagulation. Multifunctional proinflammatory cytokine
VCAM1	Vascular cell adhesion molecule 1	Cell–cell recognition, mediates leukocyte-endothelial cell adhesion
VEGFA	Vascular endothelial growth factor A	Vascular endothelial cell proliferation and migration. Angiogenesis

**Table 2:** DNA oligonucleotide sequences used for RT-qPCR.

Gene Name	Sequence (5′→3′)	Length	Reference
ADAMTS13	ATGTCGTGGCTGGGAAGATG	20	NM_139026.6
	GCCATACCGCCTGTAAACCT	20	
ALOX5	GAGAAGCACCTGCTGGACAA	20	NM_000698.5
	CGTCCACAGTCACGTCGTAT	20	
B2M	AGGACTGGTCTTTCTATCTCTTGT	24	NM_004048.4
	ACCTCCATGATGCTGCTTACA	21	
CD31	GTGCAGTACACGGAAGTTCAAG	22	NM_000442.5
	TTTCCACGGCATCAGGGACA	20	
CD44	CAGCAAACAACACAGGGGTG AGGTGGAGCTGAAGCATTGA	20 20	NM_001202555.2
EDN1	AACCAGGTCGGAGACCATGA TCACCAATGTGCTCGGTTGT	20 20	NM_001168319.2
GPX1	ACCCGGCACTTTATTAGTGGG TACGAGGGAGGAACACCTGAT	21 21	NM_001329503.2
HYAL2	CCAGTCTACGTCTTCACA GCACTCTCGCCAATGGTA	18 18	NM_033158.4
ICAM1	CAGTCAGATACAACAGCATTTGGG ACTACAGATCAGATGCGTGGC	24 21	NM_000201.3
LGALS8	CTCCAATCGACAAGAAGCTGG GAATGGTGCCAACAAACGGG	21 20	NM_201544.4
MMP1	GAAGCTGCTTACGAATTTGCC AACAGCCCAGTACTTATTCCCT	21 22	NM_002421.4
MMP2	TGCTGAAGGACACACTAAAGAAGA TCCGCATGGTCTCGATGGTA	24 20	NM_004530.6
MMP9	CTGCAACGTGAACATCTT CTCAGAGAATCGCCAGTA	18 18	NM_004994.3
NOS3	ATGAGCACTGAGATCGGCAC GTCTTTCCACAGGGACGAGG	20 20	NM_000603.5
PI16	TGCACATGAGATGGGACGAG AGGTTGTAGTGCTCACGCTC	20 20	NM 153370.3
PLA2G7	CTTGGAACACACTGGCTTATGG TGCAGGAGTTGTCATTGAACC	22 21	NM_005084.4
SOD1	TGCAGGTCCTCACTTTAATCCTC AGTCACATTGCCCAAGTCTCC	23 21	NM_000454.5
TFRC	AGCATTCCCGAAATCTGTTGT GGCCTGAGTTTACAGTGGCT	21 20	NM_003234.4
TIMP1	TTCTGCAATTCCGACCTCGT GCTGGTATAAGGTGGTCTGGT	20 21	NM_003254.3
TNF	CCGACTATCTCGACTTTGCC GATGTTCGTCCTCCTCACAG	20 20	NM_000594.4
VCAM1	CAGGCTGGAAGAAGCAGAAAG TGTCTCCTTCTTTGACACTCTCAG	21 24	NM_001078.4
VEGFA	ATCCAATCGAGACCCTGGTG AGGATGGCTTGAAGATGTACTCG	20 23	NM_001025366.3

**Table 3:** Baseline characteristics of the study population.

	INOCA (*n* = 18)	ObCCS (*n* = 20)	*p*-Value
Demographic characteristics			
Age, yrs	61 ± 9	69 ± 9	0.01 *
Sex, male/female	14/4	15/5	0.57
BMI (kg/m^2^)	27 ± 3	27 ± 2	0.67
Cardiovascular risk factors			
Hypertension (%)	17 (94)	17 (85)	0.61
Dyslipidemia (%)	12 (67)	16 (80)	0.47
Smoke (%)	12 (67)	13 (65)	0.59
Family history of IHD (%)	8 (44)	11 (55)	0.75
Obesity (%)	3 (17)	2 (10)	0.65
Diabetes (%)	5 (28)	3 (15)	0.44
History			
Previous ACS (%)	2 (11)	4 (20)	0.56
Previous PCI (%)	0 (0)	12 (60)	<0.001 *
Previous CABG (%)	0 (0)	1 (5)	0.53
Medications (at the time of blood sampling)			
Aspirin (%)	13 (72)	19 (95)	0.08
P2Y_12_ receptor inhibitors (%)	3 (18)	12 (63)	0.01 *
ACE inhibitors (%)	6 (33)	7 (35)	1
ARBs (%)	5 (28)	7 (35)	0.73
Calcium-channel blockers (%)	2 (18)	2 (10)	0.68
Statins (%)	10 (56)	19 (95)	0.01 *
β-Blockers (%)	9 (50)	16 (80)	0.09
Diuretic agents (%)	5 (28)	2 (10)	0.22
Oral antidiabetic drugs (%)	4 (22)	2 (10)	0.40
Anticoagulant drugs (%)	0 (0)	1 (5)	1
Insulin (%)	1 (6)	1 (5)	1
Laboratory assay			
cTnI > 0.004 ng/mL	0 (0)	0 (0)	NA
Haemoglobin, g/dL	13.3 ± 3.5	14.1 ± 1.5	0.73
Lymphocyte count, 10^9^/l	2.1 ± 0.8	2.3 ± 0.8	0.70
Platelets, 10^3^/mL	230 ± 66	212 ± 33	0.30
Glycemia, mg/dL	92 ± 13	92 ± 18	0.29
Total cholesterol, mg/dL	160 ± 27	150 ± 31	0.31
LDL, mg/dL	90 ± 24	88 ± 24	0.86
HDL, mg/dL	49 ± 8	44 ± 11	0.18
Triglycerides, mg/dL	104 ± 33	128 ± 60	0.17
Creatinine, mg/dL	0.89 ± 0.16	0.95 ± 0.18	0.27
hs-CRP, mg/L	3 ± 3	7.2 ± 1.8	0.72
In-hospital management			
Multivessel disease (%)	0 (0)	16 (80)	<0.001 *
LVEF ≥ 50% (%)	18 (100)	19 (95)	1
PCI for index event	0 (0)	15 (75)	<0.001 *
CABG for index event	0 (0)	3 (15)	0.23
OMT for index event	18 (100)	2 (10)	<0.001 *

ACE = angiotensin-converting enzyme; ACS = acute coronary syndromes; ARBs = angiotensin II receptor blockers; BMI = body mass index; CABG = coronary artery bypass grafting; cTnI = cardiac troponin I; HDL = high-density lipoprotein; hs-CRP = high-sensitivity C-reactive protein; INOCA = ischemia with non-obstructive coronary artery; IHD = ischemic heart disease; LDL = low-density lipoprotein; LVEF = left ventricular ejection fraction; NA = not available; ObCCS = obstructive chronic coronary syndrome; OMT = optimal medical treatment; PCI = percutaneous coronary intervention. Values are mean ± SD, *n*, *n* (%). Statistical significance (*) *p*-value < 0.05. Therapies refer to the time of blood withdrawal.

**Table 4:** Sensitivity and specificity report for different HYAL2 gene expression cut-offs.

Cut-Point	Sensitivity	Specificity	Correctly Classified	LR+	LR−
≥0.1807	100.00%	0.00%	42.86%	1.0000	
≥0.1898	100.00%	6.25%	46.43%	1.0667	0.0000
≥0.1954	100.00%	12.50%	50.00%	1.1429	0.0000
≥0.2261	100.00%	18.75%	53.57%	1.2308	0.0000
≥0.2717	100.00%	25.00%	57.14%	1.3333	0.0000
≥0.2732	100.00%	31.25%	60.71%	1.4545	0.0000
≥0.3037	100.00%	37.50%	64.29%	1.6000	0.0000
≥0.3062	100.00%	43.75%	67.86%	1.7778	0.0000
≥0.3530	100.00%	50.00%	71.43%	2.0000	0.0000
≥0.3617	100.00%	56.25%	75.00%	2.2857	0.0000
≥0.4059	100.00%	62.50%	78.57%	2.6667	0.0000
≥0.4277	100.00%	68.75%	82.14%	3.2000	0.0000
≥0.4572	100.00%	75.00%	85.71%	4.0000	0.0000
≥0.4675	100.00%	81.25%	89.29%	5.3333	0.0000
≥0.4715	100.00%	87.50%	92.86%	8.0000	0.0000
≥0.5896	100.00%	93.75%	96.43%	16.0000	0.0000
≥0.6289	91.67%	93.75%	92.86%	14.6667	0.0889
≥0.681622	83.33%	93.75%	89.29%	13.3333	0.1778
≥0.7401	75.00%	93.75%	85.71%	12.0000	0.2667
≥0.8326	66.67%	93.75%	82.14%	10.6667	0.3556
≥0.9196	58.33%	93.75%	78.57%	9.3333	0.4444
≥1.209	50.00%	93.75%	75.00%	8.0000	0.5333
≥2.020	41.67%	93.75%	71.43%	6.6667	0.6222
≥2.179	41.67%	100.00%	75.00%		0.5833
≥2.62764	33.33%	100.00%	71.43%		0.6667
≥2.69799	25.00%	100.00%	67.86%		0.7500
≥3.249	16.67%	100.00%	64.29%		0.8333
≥4.971	8.33%	100.00%	60.71%		0.9167
>4.971	0.00%	100.00%	57.14%		1.0000

## Data Availability

Data supporting the findings of this study are available from the corresponding author on request.
